# A Scoring System Based on Diffusion Tensor Imaging and Blood Biochemistry Tests for Diagnosing Biliary Atresia in Infants

**DOI:** 10.3390/children12070877

**Published:** 2025-07-03

**Authors:** Bo Liu, Xiaoying Ni, Jin Zhu, Shuang Ding, Helin Zheng, Daisong Liu, Hongrong Xu, Jinhua Cai

**Affiliations:** 1 Chongqing Key Laboratory of Pediatric Metabolism and Inflammatory Diseases, Ministry of Education Key Laboratory of Child Development and Disorders, National Clinical Research Center for Child Health and Disorders, Department of Radiology, Children’s Hospital of Chongqing Medical University, Chongqing 400014, China; 400237@hospital.cqmu.edu.cn (B.L.); 486712@hospital.cqmu.edu.cn (S.D.); 400632@hospital.cqmu.edu.cn (H.Z.); 480715@hospital.cqmu.edu.cn (D.L.); 483185@hospital.cqmu.edu.cn (H.X.); 2 Department of Radiology, The Third Affiliated Hospital of Chongqing Medical University (FangDa Hospital), Chongqing 401120, China; 652547@hospital.cqmu.edu.cn; 3 Chongqing Key Laboratory of Pediatric Metabolism and Inflammatory Diseases, Ministry of Education Key Laboratory of Child Development and Disorders, National Clinical Research Center for Child Health and Disorders, Department of Pathology, Children’s Hospital of Chongqing Medical University, Chongqing 400014, China; 102625@cqmu.edu.cn

**Keywords:** biliary atresia, magnetic resonance imaging, infant, biochemistry

## Abstract

Objectives: The aim of this study was to investigate the diagnostic value of a scoring system based on diffusion tensor imaging (DTI) and blood biochemistry tests for biliary atresia (BA) in infants. Methods: Seventy-four patients who had undergone DTI and blood biochemistry tests were included in this study. Among them, 51 (36 BA patients and 15 non-BA patients) were assigned to the training cohort, and 23 (14 BA patients and 9 non-BA patients) were assigned to the validation cohort. The characteristics that significantly differed between the groups in the training cohort were used to develop a scoring system for predicting the presence or absence of BA through binary logistic regression analysis. The scoring system was subsequently validated in the validation cohort, and its diagnostic performance was assessed with receiver operating characteristic curve analysis. Results: The mean apparent diffusion coefficient values of the hepatic right and caudate lobes and the serum levels of gamma glutamyl transpeptidase were selected for constructing the scoring system. The accuracy, sensitivity, and specificity of the system in predicting BA were 82.35%, 91.67% and 60%, respectively, in the training cohort and 95.65%, 100% and 88.89%, respectively, in the validation cohort. The areas under the receiver operating characteristic curve in the training cohort and validation cohort for predicting BA were 0.87 and 0.94 (*p* ≤ 0.001 each), respectively. Conclusions: We developed a relatively noninvasive scoring system for diagnosing BA according to the results of DTI and blood biochemistry tests, which demonstrated good performance and may be a potential method for differentiating BA in infants.

## 1. Introduction

Biliary atresia (BA) is a congenital disease characterized by partial or total atresia of the intra- and/or extrahepatic bile ducts and is the most common cause of obstructive jaundice in infants [1,2]. In BA patients, spontaneous destruction and inflammatory reactions of the intra- and extrahepatic bile ducts lead to bile duct fibrosis and obstruction [1,3]. If left untreated, BA can progress to cirrhosis, liver failure, and even death within two years [2]. Currently, the preferred method for clinically treating BA is the Kasai procedure, which restores bile flow via hepatic portoenterostomy. An important factor affecting the success of the Kasai procedure is the age at surgery [4]. Early diagnosis of BA can lead to better treatment outcomes, but making early diagnoses is challenging [5,6,7,8].

Existing methods for diagnosing BA can be divided into invasive and noninvasive methods. Invasive methods mainly include surgical exploration, laparoscopic exploration, intraoperative cholangiography (IC), liver biopsy (LB) and blood biochemistry tests, of which IC is often used as the gold standard for diagnosing BA [4]. Noninvasive methods primarily include magnetic resonance imaging (MRI), hepatobiliary scintigraphy, ultrasound and color Doppler ultrasound [9,10,11,12,13]. However, hepatobiliary scintigraphy uses radiopharmaceuticals, which are radioactive, and the results are influenced by the serum bilirubin level. MRI does not involve radiation and is especially suitable for children, but its accuracy in diagnosing BA with a single sequence, such as three-dimensional magnetic resonance cholangiopancreatography, diffusion-weighted imaging or diffusion tensor imaging (DTI), is currently insufficient [14,15,16]. The accuracy of ultrasound, especially color Doppler ultrasound, in diagnosing BA is subject to the experience of the operator [11,17]. Furthermore, blood biochemical tests lack specificity in diagnosing BA. Rendón-Macías et al. [18] suggested that gamma glutamyl transpeptidase (GGT) cannot be used alone in the diagnosis of BA. Other researchers have suggested that elevated levels of serum matrix metalloproteinase-7 (MMP-7) have significant diagnostic value for BA and could serve as a reliable biomarker for its diagnosis. However, measuring MMP-7 levels requires specialized equipment and careful consideration of factors such as the choice of ELISA kits, sample storage conditions, and experimental protocols [19,20,21]. Recently, El-Guindi et al. [22] adapted both invasive and noninvasive methods to design and validate a scoring system based on clinical, laboratory, ultrasound and histopathological characteristics for predicting BA. The scoring system consists of twelve characteristics, some of which, such as the triangular cord sign, hepatic subcapsular flow, ductular proliferation and bile plugs, need to be assessed by experienced operators. However, there are few studies on the application of a simple scoring system based on relatively noninvasive methods alone, especially MRI, for diagnosing BA.

In this study, a scoring system based on DTI and blood biochemistry tests was established and validated for diagnosing BA. Our aim was to investigate the feasibility of this scoring system for early and accurate diagnosis of BA in infants.

## 2. Materials and Methods

### 2.1. Study Population

**Figure 1:**
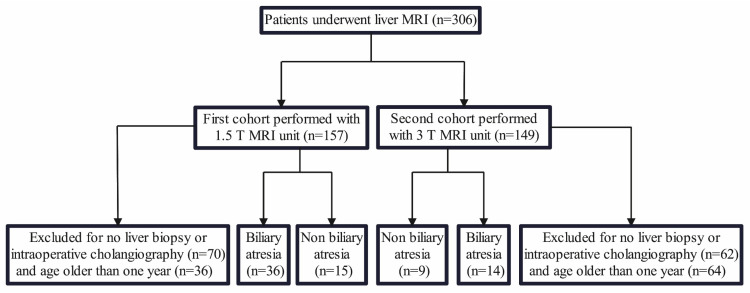
Selection of the study population. The numbers in parentheses in the boxes in the first two rows indicate the number of children who underwent liver diffusion tensor imaging. The numbers in the boxes in the third row indicate the number of children who were excluded or included.

This study included two consecutive cohorts of infants with a clinical suspicion of BA. From February 2012 to January 2024, a total of 306 patients were recruited from the Department of Hepatobiliary Surgery, Children’s Hospital of Chongqing Medical University. All patients underwent liver DTI. Among the 306 patients, 157 patients (51.3%) whose MRI data were obtained with a 1.5 T MRI unit composed the first cohort, and the other 149 patients (48.7%), whose MRI data were acquired with a 3T MRI unit, composed the second cohort. The inclusion criterion was presentation with more than one clinical symptom, such as jaundice, clay stool, hepatomegaly or dark urine. The exclusion criteria included age older than one year, no previous IC or LB, no previous blood biochemistry testing, or a follow-up of less than six months. Among these patients, 100 (32.7%) who were older than one year and 132 (43.1%) without LB or IC information were excluded. Finally, 74 patients (24.2%), comprising 39 males and 35 females, were included. Their ages ranged from 20 days to 272 days, with a median of 70 days. A flowchart depicting the recruitment of the study population is shown in Figure 1. The first cohort, whose data were used to train the scoring system, consisted of 51 patients (26 males and 25 females; median age, 67 days). The second cohort, whose data were used to validate the scoring system, consisted of 23 patients (13 males and 10 females; median age, 75 days).

### 2.2. Imaging Data Collection and Postprocessing

Twenty minutes before the MRI examination, all patients were sedated by oral administration of 10% chloral hydrate (made at the Children’s Hospital of Chongqing Medical University, Chongqing, China) at a dose of 0.5 mL/kg or by experienced anesthesiologists with a combination of intranasal dexmedetomidine and inhalation of low-dose sevoflurane [23].

The patients in the training cohort were imaged with a 1.5 T MRI unit (Signa Propeller HD; GE Medical Systems; Milwaukee, WI, USA) equipped with a single-channel quadrature head coil. The validation cohort was imaged with a 3T MRI unit (Achieva; Philips; Eindhoven, The Netherlands) equipped with a sixteen-channel phased array abdominal coil. All patients were placed in the supine and feet-first positions. Routine axial T1- and T2-weighted imaging was performed first. Then, coronal magnetic resonance cholangiopancreatography images were obtained as described in detail previously [14]. Finally, axial DTI was performed with a spin-echo single-shot echo-planar imaging sequence with the following parameters: repetition time, 4000 ms; echo time, 80 ms; matrix, 128 × 128; slice thickness, 5 mm; slice gap, 0 mm; number of excitations, 2; application of a sensitive diffusion gradient field in 15 directions; and b-factors of 0 and 1000 s/mm^2^ in the training cohort and 0 and 600 s/mm^2^ in the validation cohort. The field of view covered the diaphragmatic dome to the inferior hepatic margin.

**Figure 2:**
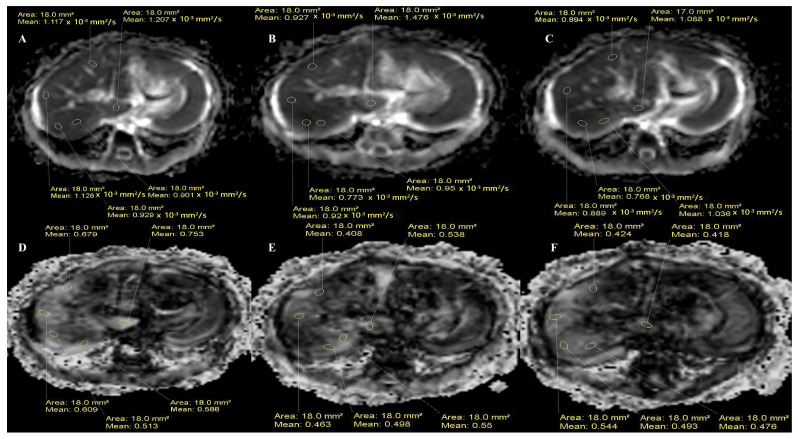
Measurement of fractional anisotropy (FA) and apparent diffusion coefficient (ADC) on diffusion tensor images in a 124-day-old female with biliary atresia using 3T MRI. Three regions of interest (ROIs) were placed on the right hepatic lobe, one ROI was placed on the left and caudate lobes each, and this process was repeated on three consecutive slices at the porta hepatis level. The mean ADC and FA values for the right lobe were calculated from nine ROIs, whereas the left and caudate lobes used three ROIs each. Measurements were performed on ADC (**A**–**C**) and FA (**D**–**F**) maps for quantitative analysis.

Using Functool postprocessing software version 9.4.05a on a workstation (ADW4.4; GE Medical Systems; Milwaukee, WI, USA) in the training cohort and diffusion postprocessing software on a separate workstation (EWS2.6.3; Philips; Eindhoven, The Netherlands) in the validation cohort, fractional anisotropy (FA) and apparent diffusion coefficient (ADC) maps were automatically calculated from the original DTI data. While attempting to avoid interference from the surrounding abdominal wall and vascular and biliary structures, five regions of interest (ROIs) were placed on the right (three ROIs), caudate (one ROI) and left hepatic lobes (one ROI) on the ADC and FA maps, from which the ADC and FA values were automatically calculated. For all the ROIs, the regions were drawn on three consecutive slices above and below the porta hepatis of each patient [15,16]. The mean values of the nine total ROIs on the right hepatic lobe were recorded as the final ADC and FA values of the right hepatic lobe for each patient (Figure 2). Similarly, the mean values of the three ROIs on the left and caudate hepatic lobes were recorded as the final ADC and FA values of the left and caudate hepatic lobes for each patient, respectively (Figure 2). The results of the above procedures were interpreted in consensus by two pediatric radiologists with fifteen and twenty years of experience in abdominal MRI who were blinded to the clinical information.

### 2.3. Blood Biochemistry Test

Blood biochemistry tests were performed one week before surgery. The results included the serum levels of GGT, total bilirubin, direct bilirubin, indirect bilirubin, aspartate aminotransferase, and alanine aminotransferase, which were measured using a fully automatic biochemical analyzer (Cobas^®^ 8000 C701; Roche Diagnostics; Basel, Switzerland). Additionally, platelet counts were determined with a fully automatic blood cell analyzer (XS-500i; SYSMEX; Kobe, Japan).

### 2.4. Histopathological Evaluation

The average time from MRI scheduling to its completion and interpretation was approximately 2 days (range 1–3 days). All patients with clinical and MRI-based suspicion of BA underwent IC or LB an average of 3 days (range 1–7 days) after the MRI examination. All samples were evaluated by an experienced pathologist. BA was diagnosed on the basis of the presence of bile duct proliferation and bile plugs [16].

### 2.5. Development of the Scoring System

First, all patients were divided into a BA group and a non-BA group according to the pathologists’ comments on the IC or LB samples or the clinical outcome. Second, the characteristics of the BA and non-BA groups in the training cohort were statistically analyzed with the Mann–Whitney U test, independent samples *t*-test, or the chi-square test. Third, in the training cohort, binary logistic regression analyses were performed to predict the presence of BA using the final confirmed diagnosis as the dependent variable and the individual characteristics with significant differences between the groups as the independent variables. Finally, all of the characteristics with significant between-group differences and their corresponding regression coefficients and constants were used to establish the scoring system.

In this study, the primary outcome was the development of a scoring system based on characteristics that significantly differed between groups, while the secondary outcomes included the diagnostic efficacy of individual imaging and biochemical characteristics.

### 2.6. Assessment of the Scoring System

To verify the feasibility of the scoring system, the data of the validation cohort of patients with a clinical suspicion of BA were applied to the system. The score of each patient in the cohort was calculated, and the predicted diagnosis and the final confirmed diagnosis were compared.

### 2.7. Statistical Analysis

Statistical analyses were performed with Statistical Product and Service Solutions version 26 (IBM Corporation; Chicago, IL, USA). The normality of the numeric data and homogeneity of variance were assessed with the Shapiro–Wilk test and the Levene test, respectively. If the data were normally distributed with equal variances, an independent samples *t*-test was used to compare the groups, with the results expressed as the means ± standard deviations. Conversely, for data that did not meet these assumptions, the Mann–Whitney U test was applied, with results reported as medians and interquartile ranges. Categorical data, expressed as the number of individuals with different conditions, were compared using the chi-square test. *p* < 0.05 was considered to indicate statistical significance. The diagnostic performance was expressed as accuracy, sensitivity, specificity, positive predictive value (PPV), and negative predictive value (NPV). The overall accuracy of the scoring system in diagnosing BA was determined from the receiver operating characteristic curve.

## 3. Results

### 3.1. Surgical and Pathological Results

In the training cohort, 36 patients were diagnosed with BA, including 4 with type I BA, 2 with type II BA, and 30 with type III BA, and 15 patients were diagnosed with non-BA diseases, including 6 with infant hepatitis, 5 with biliary stenosis, 1 with a common bile duct cyst and 3 with other diseases. In the validation cohort, 14 patients were diagnosed with BA, including 2 type I BA, 1 type II BA, and 11 type III BA, and 9 patients were diagnosed with non-BA diseases, including 2 with infant hepatitis, 1 with biliary stenosis, 2 with a common bile duct cyst and 4 with other diseases.

### 3.2. Comparison of Characteristics Between the BA and Non-BA Groups in the Training and Validation Cohorts

In both the training and validation cohorts, the mean and median ADC of the right hepatic lobe (ADC _RHL_) values were significantly lower in the BA group than in the non-BA group (*p* = 0.001 and 0.01, respectively), whereas the median ADC of the left hepatic lobe (ADC _LHL_) value was not significantly different between the two groups. In addition, in the training cohort, the mean ADC of the caudate hepatic lobe (ADC _CHL_) value was significantly lower in the BA group than in the non-BA group (*p* = 0.02). According to the biochemical analyses, in both the training and validation cohorts, the median serum level of GGT was significantly greater in the BA group than in the non-BA group (*p* < 0.001 and *p* = 0.01, respectively). There was no statistically significant difference in the demographic characteristics between the BA and non-BA groups in either the training or the validation cohorts (Table 1).

### 3.3. Diagnostic Performance of Individual Characteristics

**Figure 3:**
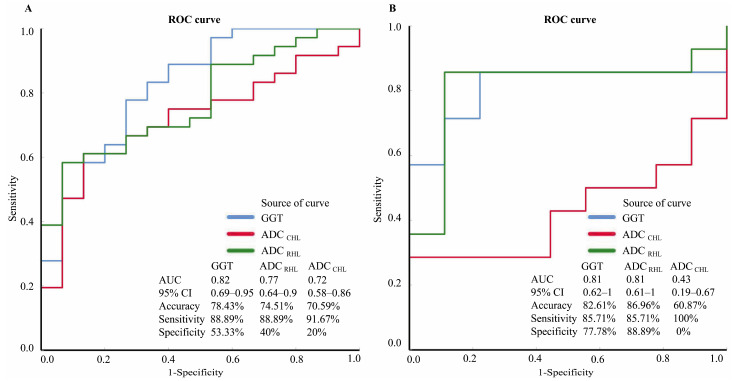
Diagnostic performance of individual characteristics for detecting biliary atresia in the training and validation cohorts. In the validation cohort (**B**), both the serum gamma glutamyl transpeptidase (GGT) level (blue line) and the apparent diffusion coefficient of the right hepatic lobe (ADC _RHL_) (green line) were more accurate and specific than those in the training cohort (**A**), but they were not as sensitive. Conversely, the apparent diffusion coefficient of the caudate hepatic lobe (ADC _CHL_) (red line) in the training cohort (**A**) had superior accuracy, specificity, and area under the receiver operating characteristic curve (AUC) compared with those in the validation cohort (**B**) but lower sensitivity. Note: ROC, receiver operating characteristic; CI, confidence interval.

The diagnostic performance of the individual characteristics with significant differences between the BA and non-BA groups was evaluated in both the training and validation cohorts (Table 2). In the training cohort, the serum GGT level had the highest performance, with an accuracy of 78.43%, followed by the mean ADC _RHL_ value (74.51%) and the mean ADC _CHL_ value (70.59%). However, in the validation cohort, the median ADC _RHL_ value had the highest performance, with an accuracy of 86.96%, followed by the serum GGT level (82.61%) and the median ADC _CHL_ value (60.87%). None of the individual characteristics had good accuracy, sensitivity, specificity, PPV, NPV, or area under the receiver operating characteristic curve (AUC) in discriminating BA patients from non-BA patients (Figure 3).

### 3.4. Performance of the Scoring System

**Figure 4:**
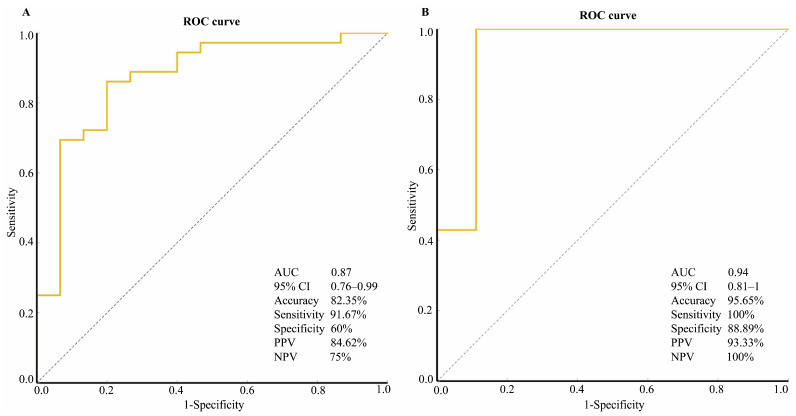
Performance of the scoring system in detecting biliary atresia in the training and validation cohorts. The overall diagnostic performance of the scoring system in discriminating biliary atresia in the validation cohort (**B**) was better than that in the training cohort (**A**) in terms of accuracy, sensitivity, specificity, positive predictive value (PPV), negative predictive value (NPV) and area under the receiver operating characteristic curve (AUC). Note: ROC, receiver operating characteristic; CI, confidence interval.

In the training cohort, when the GGT and the mean ADC values of the right and caudate lobes were used together, the diagnostic performance in detecting BA improved, yielding an accuracy of 82.35%, a sensitivity of 91.67%, a specificity of 60%, a PPV of 84.62%, an NPV of 75% and an AUC of 0.87 (Table 3, Figure 4). Therefore, we developed the following scoring system for diagnosing BA: Z = [7426.87 × ADC _RHL_ (×10^−3^ mm^2^/s) − 1454.41 × ADC _CHL_ (×10^−3^ mm^2^/s) − 6.39 × GGT (IU/L) − 3072.47]/1000. The diagnostic score was then calculated as 1/(1 + e^−Z^). If the diagnostic score was less than 0.5, the patient was diagnosed with BA; in contrast, if the score was greater than or equal to 0.5, the patient was diagnosed with non-BA.

The scoring system was then applied to the infants in the validation cohort, which included those with BA and other infantile liver diseases. All of the patients with BA (14/14) and only one (1/9) patient with biliary stenosis had a score less than 0.5 (Table 4). The diagnostic performance metrics of the scoring system in discriminating BA patients from non-BA patients included an accuracy of 95.65%, a sensitivity of 100%, a specificity of 88.89%, a PPV of 93.33%, an NPV of 100% and an AUC of 0.94 (Table 3, Figure 4).

## 4. Discussion

Owing to the limited value of a single diagnostic procedure in diagnosing BA early, a new strategy combining multiple diagnostic methods may be necessary. In this study, for the first time, we developed and validated a relatively noninvasive scoring system composed of laboratory and DTI variables for discriminating BA patients from those with other liver disorders. At a score cutoff value of 0.5, the system predicted whether the patients in the training cohort had BA with an accuracy of 82.35%, a sensitivity of 91.67%, and a specificity of 60%. Moreover, in the validation cohort, the accuracy was 95.65%, the sensitivity was 100%, and the specificity was 88.89%. The scoring system can be used as an adjunct to other noninvasive methods in the differential diagnosis of BA and non-BA diseases in infants, thus reducing the use of unnecessarily invasive examinations such as IC and LB.

In our study, the median serum levels of GGT in the BA group were greater than those in the non-BA group in both the training and validation cohorts, which is consistent with previously reported results [19,20,21,24,25,26]. The diagnostic performance of the serum level of GGT in discriminating BA patients from non-BA patients in the validation cohort was slightly better than that reported by El-Guindi et al. [22], with higher accuracy and sensitivity (82.61% vs. 78.4% and 85.71% vs. 76.7%, respectively) but lower specificity (77.78% vs. 80%). This may be because the serum level of GGT in the BA group in our study was greater than that in the aforementioned study (570.4 IU/L vs. 411.46 IU/L), and the BA group in our study was older than that in the previous study (82 days vs. 63.7 days). Some scholars have indicated that serum GGT values in patients with BA tend to increase with age [18]. Owing to progressive obstruction over the course of BA, the characteristics of the bile duct also progressively change. Increasing serum levels of GGT could therefore be an expression of the chronic inflammatory reactions that emerge in obstructed channels.

DTI parameters have been described as useful indicators for diagnosing BA. In our study, the mean and median ADC _RHL_ values in the BA group were significantly lower than those in the non-BA group in both the training and validation cohorts, which is consistent with the findings of previous studies [15,24,27]. The diagnostic performance of the median ADC _RHL_ value for BA in the validation cohort of our study was better than that in two previous studies [15,24]; specifically, our study demonstrated higher sensitivity and specificity (85.71% vs. 45.6% and 75%, 88.89% vs. 80.4% and 81.5%, respectively). This may be because the median ADC _RHL_ value in the BA group in our study was lower than that in the two previous studies (0.64 × 10^−3^ mm^2^/s vs. 1.1 × 10^−3^ mm^2^/s and 1.26 × 10^−3^ mm^2^/s, respectively). As biliary duct fibrosis and liver cirrhosis progress in these patients, the volume and function of the right hepatic lobe gradually decrease, possibly because of compression of the hepatic intercellular spaces, the development of hepatocytic inflammatory edema and fibrous bands and decreased perfusion [15,28,29]. Compression of hepatic intercellular spaces and swelling of hepatocytes can change the distribution of intra- and extracellular water [29], and the resulting restriction in water molecule diffusion may result in decreased ADC values in BA patients. In addition, some scholars have suggested that the reproducibility and reliability of the ADC _LHL_ value are lower than those of the ADC _RHL_ value [29]. Hence, we modified the method for calculating the mean ADC _RHL_ value in the scoring system. Moreover, the mean ADC _CHL_ value was significantly lower in the BA group than in the non-BA group in the training cohort, but there was no significant difference between the two groups in the validation cohort. Some scholars have suggested that hepatic ADC values in healthy adults are affected by factors such as field strength, machine vendor and DTI parameters [16,29,30,31]. In the validation cohort in this study, DTI was performed with a Philips 3T MRI unit with b values of 0 and 600 s/mm^2^; however, in the training cohort, DTI was performed with a GE 1.5 T MRI unit with b values of 0 and 1000 s/mm^2^. This may have caused the difference in the ADC _CHL_ values observed between the training and validation cohorts. In addition, the significant difference in the ADC _CHL_ value between the two groups in the training cohort but not the validation cohort may also be related to the small sample size of the latter. Further studies with the same DTI parameters, field strengths, machine vendors and larger numbers of patients are needed to validate the effectiveness of the ADC _CHL_ value in distinguishing BA patients from non-BA patients.

The diagnostic performance of our scoring system is slightly inferior to that of the system established by El-Guindi et al. [22] for discriminating BA, with accuracies of 95.65% vs. 98.83%, specificities of 88.89% vs. 97.67%, respectively, and the same sensitivity (100%). Among the individual characteristics in the previously reported scoring system, hepatic subcapsular flow achieved the highest score, but this characteristic needs to be assessed by experienced and trained operators. The second and third highest scores were obtained with ductular proliferation and bile plugs, which first require the patient to undergo LB. However, LB is an invasive examination that has several disadvantages, including sampling error, subjectivity and bleeding complications, especially in young children [29,32]. In contrast, our scoring system consists of only three objective parameters based on laboratory examinations and DTI, which can be easily obtained relatively noninvasively.

Only one patient in the validation cohort who actually had a non-BA disease according to the IC was incorrectly predicted to have BA by the scoring system. Compared with the other non-BA patients, the misdiagnosed patient had an obviously lower ADC _RHL_ value but a greater ADC _CHL_ value and a greater serum GGT level. This could have resulted in a low diagnostic score of 0.03, resulting in a misdiagnosis.

Furthermore, compared with that of single imaging indicators such as the ADC or FA, the performance of this scoring system in diagnosing BA was better in terms of accuracy, sensitivity, specificity, PPV, NPV and AUC [16]. Therefore, our scoring system could be a simple, noninvasive screening method for discriminating infants with BA from those with non-BA liver diseases.

In both the training and validation cohorts, there was a significant difference in the mean and median ADC _RHL_ values between the BA and non-BA groups. However, no significant difference in the median ADC _LHL_ value was found between the two groups. This may be due to the good regenerative ability of the left hepatic lobe, which makes this part of the liver less prone to fibrosis than the right lobe [33,34]. Hepatic fibrosis leads to excessive synthesis of the extracellular matrix, especially collagen fibers [35,36,37], which can restrict the diffusion of water molecules, resulting in a decrease in the ADC value [38]. Moreover, because hepatic cirrhosis is prominent in BA patients, the affected hepatic parenchyma is usually grossly deformed [39,40]. As liver cirrhosis progresses, the right hepatic lobe tends to shrink further [28]; hence, water molecule diffusion in the right hepatic lobe is more restricted than that in the left hepatic lobe. Therefore, in our study, the mean and median ADC _RHL_ values in the BA group were significantly lower than those in the non-BA group, even across different field strengths, machine vendors and DTI parameters. However, the median ADC _LHL_ value did not differ significantly between the two groups. In future studies, the ADC differences between the right and left hepatic lobes in BA patients should be further validated with a larger sample size.

This study has several limitations. First, the sample size was relatively small, especially in the validation cohort. The median age of participants in this study was 70 days, which suggests that the scoring system may not be suitable for infants younger than 60 days. Further studies with larger sample sizes are needed to validate the efficacy of our scoring system in distinguishing BA from non-BA diseases, particularly in infants under 60 days of age. Second, there was a bias from the selection of different field strengths, machine vendors and b values of DTI, which may have affected the results. In future studies, we will attempt to use the same field strengths, machine vendors and DTI parameters to explore the differences in the hepatic ADC and FA values between the BA and non-BA groups while minimizing this bias. Third, the data in our study were obtained from a single center. In the future, multicenter studies including external validation are needed. Fourth, a potential confounding factor in this study was the choice of different sedation methods, such as oral chloral hydrate or a combination of intranasal dexmedetomidine and inhaled sevoflurane. These agents differ in their sedative depth and physiological effects, which could influence motion artifacts and hepatic perfusion, potentially affecting DTI parameters such as ADC and FA. Fifth, since our scoring system was based solely on routine blood biochemistry tests and hepatic DTI parameters, other potentially useful biomarkers were not assessed. Future development of the scoring system should include additional indicators, such as serum MMP-7 levels, to improve its accuracy and reliability. Finally, all samples in this study were evaluated by a single experienced pathologist. However, in clinical studies, histopathological evaluation should be performed by at least two independent pathologists to ensure diagnostic consistency. Future studies should incorporate assessments by two independent expert pediatric pathologists to increase the reliability and validity of the findings.

## 5. Conclusions

We developed a relatively noninvasive scoring system for diagnosing BA on the basis of readily available laboratory and DTI data. While this system demonstrated high accuracy in distinguishing BA infants from non-BA infants, these findings should be interpreted with caution due to limitations such as the small sample size, single-center design, and reliance on DTI. Further validation in larger, multicenter cohorts is necessary before it can be broadly applied in clinical settings, especially in infants under 60 days of age.

## Figures and Tables

**Table 1:** Laboratory, DTI and demographic characteristics of the training and validation cohorts.

	Training Cohort (n = 51)			Validation Cohort (n = 23)		
Characteristics	BA Group (n = 36)	Non-BA Group (n = 15)	*p* Value	BA Group (n = 14)	Non-BA Group (n = 9)	*p* Value
TB (μmol/L)	181.6 (147.08–211.9) ^●^	173.2 (154.6–221) ^●^	0.85 ^★^	150 (127.53–184.45) ^●^	161.8 (74.45–195.05) ^●^	0.56 ^★^
DB (μmol/L)	118.43 ± 39.98	110.6 ± 38.19	0.52 ^▲^	111.62 ± 36.77	97.28 ± 56.13	0.47 ^▲^
IB (μmol/L)	69.15 (51.33–83.05) ^●^	58.5 (42–90.8) ^●^	0.61 ^★^	50.6 (31.4–72.78) ^●^	45.4 (23–54.5) ^●^	0.37 ^★^
AST (IU/L)	209.2 (166.75–316.6) ^●^	232.4 (169.8–321.8) ^●^	0.9 ^★^	271.2 (198.85–287.33) ^●^	168.4 (124.85–244.95) ^●^	0.06 ^★^
ALT (IU/L)	155.35 (87.43–225.33) ^●^	135.2 (76.7–209) ^●^	0.43 ^★^	151.05 (116.08–233.48) ^●^	124.7 (63.95–218.8) ^●^	0.28 ^★^
GGT (IU/L)	511.55 (274.83–973.78) ^●^	170.8 (77.4–339.6) ^●^	<0.001 ^★^	570.4 (351.5–1039.73) ^●^	157.2 (126.65–329.55) ^●^	0.01 ^★^
Platelet count (×10^9^/L)	403.58 ± 140.77	412.13 ± 192.95	0.86 ^▲^	470.07 ± 196.89	382.67 ± 133.7	0.26 ^▲^
FA _LHL_	0.4 (0.33–0.47) ^●^	0.4 (0.38–0.43) ^●^	0.76 ^★^	0.54 (0.44–0.61) ^●^	0.43 (0.42–0.49) ^●^	0.05 ^★^
FA _CHL_	0.41 ± 0.12	0.4 ± 0.12	0.74 ^▲^	0.47 ± 0.1	0.43 ± 0.08	0.38 ^▲^
FA _RHL_	0.4 ± 0.07	0.41 ± 0.08	0.58 ^▲^	0.62 ± 0.11	0.63 ± 0.11	0.73 ^▲^
ADC _LHL_ (×10^−3^ mm^2^/s)	1.22 (1.12–1.51) ^●^	1.41 (1.22–1.44) ^●^	0.34 ^★^	1.3 (1.13–1.54) ^●^	1.29 (1.16–1.33) ^●^	0.83 ^★^
ADC _CHL_ (×10^−3^ mm^2^/s)	1.23 ± 0.22	1.38 ± 0.17	0.02 ^▲^	1.18 (0.95–1.48) ^●^	1.22 (1.13–1.28) ^●^	0.6 ^★^
ADC _RHL_ (×10^−3^ mm^2^/s)	1.22 ± 0.13	1.37 ± 0.13	0.001 ^▲^	0.64 (0.57–0.96) ^●^	1.11 (1.03–1.17) ^●^	0.01 ^★^
Age (days)	66.5 (54–107.8) ^●^	67 (49–82) ^●^	0.61 ^★^	82 (45–101.5) ^●^	74 (57.5–145) ^●^	0.83 ^★^
Sex	M/F (17/19)	M/F (9/6)	0.89 ^❆^	M/F (7/7)	M/F (6/3)	0.53 ^❆^

Note: DTI, diffusion tensor imaging; n, number of patients; BA, biliary atresia; TB, total bilirubin; DB, direct bilirubin; IB, indirect bilirubin; AST, aspartate aminotransferase; ALT, alanine aminotransferase; GGT, gamma glutamyl transpeptidase; FA, fractional anisotropy; LHL, left hepatic lobe; CHL, caudate hepatic lobe; RHL, right hepatic lobe; ADC, apparent diffusion coefficient; M/F, male/female; ● represents the median and interquartile range; ▲ represents the independent samples *t*-test; ★ represents the Mann–Whitney U test; ❆ represents the chi-square test.

**Table 2:** Diagnostic performance of individual characteristics for BA in the training and validation cohorts.

Characteristics	AUC	*p* Value	Accuracy	Sensitivity	Specificity	PPV	NPV
Training cohort (n = 51)							
GGT	0.82	<0.001	78.43%	88.89% (0.78–1) ^■^	53.33% (0.25–0.82) ^■^	82.05% (0.69–0.95) ^■^	66.67% (0.35–0.98) ^■^
ADC _CHL_	0.72	0.02	70.59%	91.67% (0.82–1.01) ^■^	20% (−0.03–0.43) ^■^	73.33% (0.6–0.87) ^■^	50% (−0.08–1.08) ^■^
ADC _RHL_	0.77	0.002	74.51%	88.89% (0.78–1) ^■^	40% (0.12–0.68) ^■^	78.05% (0.65–0.91) ^■^	60% (0.23–0.97) ^■^
Validation cohort (n = 23)							
GGT	0.81	0.01	82.61%	85.71% (0.65–1.07) ^■^	77.78% (0.44–1.12) ^■^	85.71% (0.65–1.07) ^■^	77.78% (0.44–1.12) ^■^
ADC _CHL_	0.43	0.57	60.87%	100% (1–1) ^■^	0% (0–0)^■^	60.87% (0.39–0.82) ^■^	N/A
ADC _RHL_	0.81	0.01	86.96%	85.71% (0.65–1.07) ^■^	88.89% (0.63–1.15)^■^	92.31% (0.75–1.09) ^■^	80% (0.5–1.1) ^■^

Note: BA, biliary atresia; AUC, area under the receiver operating characteristic curve; PPV, positive predictive value; NPV, negative predictive value; GGT, gamma glutamyl transpeptidase; ADC, apparent diffusion coefficient; CHL, caudate hepatic lobe; RHL, right hepatic lobe; N/A represents not applicable; n, number of patients; ■ represents the 95% confidence interval.

**Table 3:** Diagnostic performance of the scoring system for discriminating BA patients from non-BA patients in the training and validation cohorts.

Gold Standard	Training Cohort (n = 51)		Validation Cohort (n = 23)	
	BA	Non-BA	BA	Non-BA
BA	33	3	14	0
Non-BA	6	9	1	8
Accuracy	82.35% (42/51)		95.65% (22/23)	
Sensitivity	91.67% (0.82–1.01) ^■^ (33/36)		100% (1–1) ^■^ (14/14)	
Specificity	60% (0.32–0.88) ^■^ (9/15)		88.89% (0.63–1.15) ^■^ (8/9)	
PPV	84.62% (0.73–0.97) ^■^ (33/39)		93.33% (0.79–1.08) ^■^ (14/15)	
NPV	75% (0.46–1.04) ^■^ (9/12)		100% (1–1 )^■^ (8/8)	
AUC	0.87 (*p* < 0.001)		0.94 (*p* = 0.001)	

Note: BA, biliary atresia; PPV, positive predictive value; NPV, negative predictive value; AUC, area under the receiver operating characteristic curve; n, number of patients; ■ represents the 95% confidence interval.

**Table 4:** Performance metrics of the scoring system in individual infants and comparison with the final confirmed diagnosis in the validation cohort.

Patient Number	Diagnostic Score	Diagnosis by the Scoring System	Confirmed Diagnosis	Patient Number	Diagnostic Score	Diagnosis by the Scoring System	Confirmed Diagnosis
1	0.41	BA	Correct	13	0.97	Non-BA	Correct
2	0.4	BA	Correct	14	0.09	BA	Correct
3	0.12	BA	Correct	15	0.91	Non-BA	Correct
4	<0.001	BA	Correct	16	0.01	BA	Correct
5	<0.001	BA	Correct	17	0.08	BA	Correct
6	0.68	Non-BA	Correct	18	<0.001	BA	Correct
7	0.78	Non-BA	Correct	19	0.96	Non-BA	Correct
8	0.03	BA	Correct	20	0.07	BA	Correct
9	0.92	Non-BA	Correct	21	0.48	BA	Correct
10	0.03	BA	Incorrect	22	0.01	BA	Correct
11	0.23	BA	Correct	23	0.94	Non-BA	Correct
12	0.88	Non-BA	Correct				

Note: BA, biliary atresia.

## Data Availability

The data generated or analyzed during the study are subject to privacy/ethical/legal restrictions and cannot be made publicly available. However, the data will be provided upon reasonable request to the corresponding author, in compliance with applicable regulations and ethical guidelines.

## Abbreviations

The following abbreviations are used in this manuscript:

BA	Biliary atresia
IC	Intraoperative cholangiography
LB	Liver biopsy
MRI	Magnetic resonance imaging
DTI	Diffusion tensor imaging
GGT	Gamma glutamyl transpeptidase
MMP-7	Matrix metalloproteinase-7
FA	Fractional anisotropy
ADC	Apparent diffusion coefficient
ROI	Region of interest
PPV	Positive predictive value
NPV	Negative predictive value
RHL	Right hepatic lobe
LHL	Left hepatic lobe
CHL	Caudate hepatic lobe
AUC	Area under the receiver operating characteristic curve
TB	Total bilirubin
DB	Direct bilirubin
IB	Indirect bilirubin
AST	Aspartate aminotransferase
ALT	Alanine aminotransferase
ROC	Receiver operating characteristic
CI	Confidence interval
M	Male
F	Female
